# Prevalence, Cell Tropism, and Clinical Impact of Human Parvovirus Persistence in Adenomatous, Cancerous, Inflamed, and Healthy Intestinal Mucosa

**DOI:** 10.3389/fmicb.2022.914181

**Published:** 2022-05-24

**Authors:** Man Xu, Katarzyna Leskinen, Tommaso Gritti, Valerija Groma, Johanna Arola, Anna Lepistö, Taina Sipponen, Päivi Saavalainen, Maria Söderlund-Venermo

**Affiliations:** ^1^Department of Virology, University of Helsinki, Helsinki, Finland; ^2^Research Programs Unit, Department of Immunobiology, University of Helsinki, Helsinki, Finland; ^3^Joint Laboratory of Electron Microscopy, Riga Stradin,s University, Riga, Latvia; ^4^Department of Pathology, University of Helsinki, Helsinki, Finland; ^5^HUS Diagnostic Centre, Helsinki University Hospital, Helsinki, Finland; ^6^Department of Colorectal Surgery, Helsinki University Hospital, Helsinki, Finland; ^7^Applied Tumor Genomics, Research Programs Unit, University of Helsinki, Helsinki, Finland; ^8^HUCH Abdominal Center, Helsinki University Hospital, Helsinki, Finland; ^9^Folkhälsan Research Center, Helsinki, Finland

**Keywords:** parvovirus B19, human bocavirus 1, RNAscope *in situ* hybridization, immunohistochemistry, RNA-seq, tissue persistence

## Abstract

Parvoviruses are single-stranded DNA viruses, infecting many animals from insects to humans. Human parvovirus B19 (B19V) causes erythema infectiosum, arthropathy, anemia, and fetal death, and human bocavirus (HBoV) 1 causes respiratory tract infections, while HBoV2-4 are enteric. Parvoviral genomes can persist in diverse non-permissive tissues after acute infection, but the host-cell tropism and the impact of their tissue persistence are poorly studied. We searched for parvoviral DNA in a total of 427 intestinal biopsy specimens, as paired disease-affected and healthy mucosa, obtained from 130 patients with malignancy, ulcerative colitis (UC), or adenomas, and in similar intestinal segments from 55 healthy subjects. Only three (1.6%) individuals exhibited intestinal HBoV DNA (one each of HBoV1, 2, and 3). Conversely, B19V DNA persisted frequently in the intestine, with 50, 47, 31, and 27% detection rates in the patients with malignancy, UC, or adenomas, and in the healthy subjects, respectively. Intra-individually, B19V DNA persisted significantly more often in the healthy intestinal segments than in the inflamed colons of UC patients. The highest loads of B19V DNA were seen in the ileum and colon specimens of two healthy individuals. With dual-RNAscope *in situ* hybridization and immunohistochemistry assays, we located the B19V persistence sites of these intestines in mucosal B cells of lymphoid follicles and vascular endothelial cells. Viral messenger RNA transcription remained, however, undetected. RNA sequencing (RNA-seq) identified 272 differentially expressed cellular genes between B19V DNA-positive and -negative healthy ileum biopsy specimens. Pathway enrichment analysis revealed that B19V persistence activated the intestinal cell viability and inhibited apoptosis. Lifelong B19V DNA persistence thus modulates host gene expression, which may lead to clinical outcomes.

## Introduction

Parvoviruses are small non-enveloped single-stranded DNA viruses, infecting host-specifically a multitude of animals. In humans, the most pathogenic parvoviruses are parvovirus B19 (B19V), causing the childhood rash erythema infectiosum (fifth disease), arthropathies, acute or chronic anemias, hepatitis, and fetal myocarditis, hydrops, and death, whereas human bocavirus (HBoV) 1 causes mild to life-threatening pediatric respiratory tract infections. The closely related HBoV2-3 cause enteric infections, whereas HBoV4 is extremely rare (Qiu et al., 2017; Christensen et al., 2019). B19V and HBoV infections are common worldwide, with a 40–80% seroprevalence among adults for B19V and 80, 50, 10%, and 0–1% among children under 6 years of age for HBoV1-4, respectively (Röhrer et al., 2008; Guo et al., 2012; Kantola et al., 2015; Qiu et al., 2017).

B19V DNA has been detected in various tissue types including synovium, skin, heart, liver, muscle, tonsil, testis, brain, salivary glands, bone, and bone marrow (Cassinotti et al., 1997; Söderlund et al., 1997; Eis-Hübinger et al., 2001; Söderlund-Venermo et al., 2002; Vuorinen et al., 2002; Lotze et al., 2004; Hobbs, 2006; Norja et al., 2006; Manning et al., 2007; Adamson-Small et al., 2014; Toppinen et al., 2015a; Pyöriä et al., 2017; Santonja et al., 2017), of both symptomatic and healthy individuals. HBoV DNAs have been detected in tonsils, intestine, and heart (Kuethe et al., 2009; Kapoor et al., 2011; Proenca-Modena et al., 2012; Schildgen et al., 2013; Abdel-Moneim et al., 2016; Qiu et al., 2017; Ivaska et al., 2019; Xu et al., 2021). Associations of B19V or HBoV infection with inflammatory bowel diseases, such as ulcerative colitis (UC), or gastrointestinal tumors have been suggested, but the causative role has not been verified (Li et al., 2007; Pironi et al., 2009; Schildgen et al., 2013; Abdel-Moneim et al., 2016).

Productive infection of B19V occurs exclusively in primary erythroid progenitor cells (EPCs) of the bone marrow (Ozawa et al., 1986). A still unknown cellular surface receptor, interacting with the unique part of the minor viral capsid protein (VP1u), mediates the internalization of the virion (Leisi et al., 2013; Bieri and Ros, 2019). For a productive infection, the blood-group P antigen, globoside, is further required at a post-entry step (Brown et al., 1993; Bieri et al., 2021).

During *in vivo* persistence in non-erythroid tissues, B19V has been reported to express mRNA and protein (Adamson-Small et al., 2014; Bock, 2014; Leon et al., 2017; Alves et al., 2020), whereas evidence of productive virus replication is lacking. In B19V-infected non-permissive cell cultures, VP and NS1 mRNAs may be expressed (Zakrzewska et al., 2005; Ganaie and Qiu, 2018). In monocytes, endothelial cells, and both primary and immortalized B cells, the uptake of B19V has been shown to occur through antibody-dependent enhancement (ADE) *via* the Fc receptor or complement factor C1q (Munakata et al., 2006; von Kietzell et al., 2014; Pyöriä et al., 2017), a mechanism initially shown for Aleutian mink disease virus and recently also for HBoV1 (Kanno et al., 1993; Xu et al., 2021). The natural host cells for HBoVs are unknown, but HBoV1 is able to replicate and cause pathological changes in non-dividing polarized air-liquid cell cultures of human airway epithelia (Huang et al., 2012).

Due to the difficulty of studying viruses in human tissues, the host cells of parvovirus persistence, and how these viruses modulate host cells, are still largely unknown. Many studies have used cell cultures to characterize host responses during infection. In highly permissive EPCs, B19V can induce the cellular DNA-damage response and the DNA repair machinery, as well as cell-cycle arrest (Qiu et al., 2017; Ganaie and Qiu, 2018), B19V is thus a master in manipulating cellular pathways during acute infection. However, the impact of long-lasting B19V persistence on the host cells or tissues needs to be determined.

We aimed to study the parvovirus occurrence, sites of persistence, host cells, virus activity, and transcriptomic changes in the intestinal mucosal specimens. HBoV DNA was detected in intestinal biopsy specimens of three individuals, one each of HBoV1, 2, and 3. In sharp contrast, B19V DNA persisted in 45–50% of the intestinal specimens of patients with malignancy or UC, and in 27% of healthy individuals. The host cells with high-load B19V-DNA persistence in the intestinal mucosal tissues were identified, with RNAscope *in situ* hybridization (RISH) and immunohistochemistry (IHC), to be vascular endothelial cells and follicular B lymphocytes. By whole-transcriptome sequencing, we further observed B19V modulation of cell viability and apoptosis, perhaps favoring small-scale virus replication for viral maintenance in the tissue.

## Materials and Methods

### Patients and Samples

In total, 427 fresh-frozen intestinal biopsy specimens were obtained either during ileocolonoscopy or during colorectal cancer surgery from 185 individuals at the Helsinki University Hospital (Helsinki, Finland), in 2014–2015. Paired mucosa of the disease-affected colon and healthy segments (adjacent healthy colon or ileum or both) were obtained from 130 patients (aged 20–86 years; mean 51; median 52.3) presenting with malignancy (*N* = 16), active (*N* = 42) or inactive (*N* = 33) UC, or adenomas (*N* = 39), and from 55 healthy subjects. Active UC specimens are defined as inflamed colon mucosa with moderate or severe inflammation, whereas inactive UC specimens had no present activity but had been previously severely inflamed. The “healthy” status of an individual was determined when the absence of disease was confirmed histopathologically. Two pieces per region of fresh intestinal mucosal specimens obtained surgically, were immediately immersed in RNAlater overnight at 4°C, and then stored at −20°C.

Thirteen patients (four with inactive UC, seven with active UC, and two with adenoma) had available serum samples. Formalin-fixed and paraffin-embedded (FFPE) intestinal mucosa blocks (34 specimens from 19 individuals), obtained from the same regions of the same individuals as the RNAlater-stored samples, were furthermore obtained from Helsinki Biobank. The study was approved by the Ethics Committee of the HUS Helsinki University Hospital (Decision number 553/E6/01), and informed consent was obtained from all individuals participating in this study. All ethical guidelines of HUS were followed in the research conduct.

### Viral DNA qPCR

Total DNA was extracted from all mucosal biopsy specimens of the intestine with QIAamp DNA mini kit (Qiagen, Germany) following the manufacturer’s protocol. Viral DNA was detected and quantified by in-house real-time quantitative PCRs (qPCRs). Briefly, B19V DNA was detected with Pan-B19 qPCR, amplifying a 154-bp region of the *NS1* gene of the B19V genome, as described (Toppinen et al., 2015b), and the qPCR products were sequenced for genotyping. HBoV1-4 DNAs were amplified by a multiplex qPCR, and positive results were further confirmed with singleplex qPCRs, as described (Kantola et al., 2010). All samples were tested by qPCR in duplicate. The human single-copy *RNase P* gene was quantified in all samples by qPCR, and serial dilutions of the plasmid (a kind gift from Dr. Janet Bute) containing the target amplicon served as control, as described (McNees et al., 2005; Toppinen et al., 2015b). Viral DNA copies were normalized to one million human cells.

### RNA Extraction and Reverse-Transcription PCR

Total RNA was extracted, with RNeasy Mini Kit (Qiagen, Germany), from 31 B19V DNA-positive (16 healthy ileum, 2 malignant colon, 4 adenomatous colon, 3 active UC colon, and 6 healthy sigmoid colon) and 16 DNA-negative (13 ileum and 3 sigmoid colon) mucosal biopsy specimens. These specimens were matched and selected mainly for RNA-seq library preparation or had the highest B19-DNA loads. On-column digestion with RNase-free DNase (Qiagen) was applied to remove genomic DNA contamination, according to the kit manual, and extra DNase I treatment (15 min at 37°C, Promega) was applied to the RNA preps which still contained viral DNA after initial DNase treatment. RNA was reverse transcribed with M-MLV Reverse Transcriptase (Life Technologies) and random hexamers (Promega). Probe-based real-time PCRs were used to detect B19V mRNA. The following primer pairs were used to amplify spliced VP mRNAs: forward 5′-TGCTTTTTCCTGGACTTTC-3′ (540–558, GenBank no. AY386330, genotype 1) and 5′-ACTTGCTGTTATTTGCCTGCTA-3′ (548–569, GenBank no. AB550331.1, genotype 2), reverse 5′-TTCGGAGGAAACTGGGCTT-3′ (2,283–2,301, GenBank no. AY386330, targeting both genotypes 1 and 2). Two VP splice-variant transcripts can be amplified by this RT-PCR, producing amplicons of 139 and 259 nt in length. The primer design is based on a published transcription map (Qiu et al., 2017). Non-spliced NS1 mRNA was searched for with forward primer 5′-AGCAGCAGTGGTGGTGAAAG-3′ (2,143–2,162) and reverse primer 5′-TTCGGAGGAAACTGGGCTT-3′ (2,283–2,301, GenBank no. AY386330). VP and NS1 mRNA RT-PCRs use the same probe, 5′-FAM-CCCCGGGACCAGTTCAGGAG-BHQ1-3′ (2,250–2,269, GenBank no. AY386330). DNase I-treated RNA preps without reverse transcriptase served as non-RT controls. The cloned near-full-length genomes of B19 genotypes 1 (AY504945) and 2 (AY044266) were transfected into HEK293 cells, and cDNA from these cells served as positive sample control. The PCR products of target amplicons, spliced VP and non-spliced NS1, were cloned into pSTBlue vector and served as assay controls. *RNA polymerase II (RPII),* human housekeeping mRNA, was quantified for each RNA prep as internal control, as described (Xu et al., 2021).

### Serology

As blood samples were not routinely drawn from all study subjects, only 13 serum samples were available. B19V-specific IgG and IgM were measured by in-house enzyme immunoassays (EIA), based on native VP2 virus-like particles (VLPs; Söderlund et al., 1995; Kaikkonen et al., 1999; Maple et al., 2014). To distinguish acute from past B19V infection, we used an epitope type-specific (ETS) EIA, comparing linear and conformational epitopes (Söderlund et al., 1995; Kaikkonen et al., 1999). The ETS ratio was calculated from the absorbances (OD_492nm_) of conformational VP2 IgG EIA, divided by that of the linear-epitope peptide EIA, a ratio of ≤20 is considered an acute infection.

### RNA-Seq Library Preparation, Sequencing, and Read Alignment

RNA samples were quantified and analyzed for quality in a LabChip GXII Touch HT electrophoresis system (PerkinElmer). Samples with an RNA integrity number (RIN) over 8 were used for library preparation. The library was prepared based on the Drop-seq protocol (Macosko et al., 2015) with some modifications for 3′ capturing bulk RNA-seq with sample index barcode containing polyT oligos, details described by Vuorela et al. (2021). Sequencing of the libraries was performed on Illumina NextSeq 500 at the Functional Genomics Unit of the University of Helsinki, Finland. Raw reads were additionally filtered to remove polyA tails, then aligned to the human reference genome, GRCh38using STAR aligner (Dobin et al., 2013). Gene transcripts for each gene were counted by Unique Molecular Identifiers, and samples were identified by sample index barcodes.

### Differential Expression and Pathway Analysis

Differential gene expression between B19V DNA-positive (*N* = 11) and -negative ileum (*N* = 7) was conducted with DESeq 2 package on Chipster analysis software (CSC). Among all 18 normal (healthy) ileum specimens, 9 were from healthy individuals and 9 from diseased patients (3 with inactive UC, 4 with active UC, and 2 with adenoma). To identify differently expressed genes (DEGs), a Log_2_ fold-change (Log_2_ FC) for each DEG was calculated with the Wald test adjusted by Benjamini–Hochberg, and significant DEGs were determined using an adjusted value of *p* < 0.05. For pathway analysis, the DEGs were uploaded to the ingenuity pathway analysis (IPA) software (Qiagen) and the likelihood of association between a gene set and a biological pathway or process was predicted with Fischer’s exact test; a value of *p* < 0.05 was considered significant. For each biological pathway or process, a z-score is calculated based on Log_2_ FC of the DEGs to predict the directional effect; a positive z-score is predicted to be activation and a negative z-score to be inhibition of a pathway, where only z-scores >2 or < −2 are considered significant.

### RNAscope *in situ* Hybridization and Immunohistochemistry

To identify the persistence sites of B19V and HBoV, RNAscope ISH (RISH) technology (Advanced Cell Diagnostics, ACD, CA) was applied to detect viral DNA/RNA in 5 μm-thick FFPE intestinal mucosal tissue sections from 9 individuals (6 B19V positive; 1 high load, and 5 low load). Viral nucleic acids were detected using 20 pairs of double-Z oligonucleotide probes targeting the sense B19V *NS1* gene (Probe-V-B19V-NS, 967–2,215 bp; GenBank: NC_000883.2) or the antisense HBoV *NS1* gene (Probe-V-HBoV1-NS-sense, 1,152–2,267 bp; GenBank: JQ923422.1). B19V encapsidates both DNA strands at equal frequency, while HBoV prefers the negative (antisense) strand (Summers et al., 1983; Böhmer et al., 2009). For HBoV1 RISH, the sense probe, detecting the negative-sense genome strand, has further been shown to be more sensitive than the antisense probe. The hybridized probes were amplified using RNAscope 2.5 HD Reagent Kit-RED (ACD) following the manufacturer’s manual, and signals were detected with Red Chromogen. Probes targeting the human housekeeping gene *PPIB* and the bacterial gene *dapB* (ACD) served as positive and negative controls, respectively, in each assay. Sections were counterstained with hematoxylin and mounted with VectaMount medium (Vectorlabs). To differentiate B19V DNA from mRNA signals, the intestinal sections were treated with 5 mg/ml RNase A (Qiagen) for 30 min at 40°C prior to RISH.

B19V RISH-positive cell types were identified by immunohistochemistry (IHC), with B (mouse anti-human CD20 mAb, Clone L26, Biocare Medical) or endothelial (mouse anti-human CD31 mAb, Clone BC2, Biocare Medical) cell markers and visualized using MACH1 Universal HRP Polymer Detection Kit (Biocare Medical). Vina Green Chromogen Kit (Biocare Medical) was applied to distinguish the cell markers from the red RISH signal. Finally, the stained slides were scanned with a 3DHISTECH Pannoramic 250 FLASH II digital slide scanner at the Genome Biology Unit, supported by HiLIFE and the Faculty of Medicine, University of Helsinki, and Biocenter Finland. Bright-field images were generated and analyzed with the digital software, CaseViewer 2.4 (3DHISTECH).

B19V IHC was performed at the Riga Stradiņs University, Riga, Latvia. An anti-parvovirus B19 antibody (Millipore, Bilerica, MA, United States, clone R92F6, 1:150), recognizing an epitope common to VP1 and VP2 structural proteins, was applied overnight at 4°C for the detection of B19V capsid proteins. The primary antibody was amplified using the HiDef Detection HRP Polymer system (CellMarque, Rocklin, CA, United States), and the antigen sites were visualized with 3,3′-diaminobenzidine (DAB) tetrahydrochloride kit (Cell Marque, Rocklin, CA, United States). The cell nuclei were counterstained with Mayer’s hematoxylin.

### Statistical Analysis

Fischer’s exact test (two-sided) was used to compare the presence of B19V DNA in the diseased colons and the healthy tissues of each patient group, and for comparison of B19V DNA presence in diseased and healthy groups. B19V DNA loads were compared with one-way ANOVA or Student’s *t*-test using GraphPad Prism 7 software (San Diego, CA). A value of *p* < 0.05 was considered statistically significant.

## Results

### B19V DNA in Intestinal Biopsy Specimens

For clarification, the majority of patients with malignancy provided both malignant and adjacent healthy colonic specimens, and one patient also provided healthy ileum. All patients with UC or adenoma provided both disease-affected colonic and healthy ileum specimens, whereas 8 inactive UC, 22 active UC, and 19 adenoma patients also provided adjacent healthy colon specimens. All healthy individuals provided both colon and ileum specimens.

Overall, 100 out of 427 (23.4%) intestinal mucosal specimens harbored B19V DNA by Pan-B19V qPCR. Of the 16 patients with malignancy, 8 (50%) were B19V DNA positive. Two (2/8, 25%) such patients had B19V DNA in both healthy and malignant colon, and 6/8 (75%) had B19V DNA only in healthy tissue, whereas none (0%) had B19V exclusively in malignant tissues (Table 1).

**Table 1:** B19V DNA presence in the intestinal mucosal biopsy specimens in each study group.

Study group	Patients, no.	B19V+ patients, no. (%)	Mean B19V copies/10^6^ cells	B19V+/− status in patients, no.			
				Affected colon+/normal tissue+	Affected colon−/normal tissue+	Affected colon+ /normal tissue-	Affected colon−/normal tissue-
Malignancy	16	8 (50)	26	2	6	0	8
Inactive UC	33	16 (48)	162	4	11	1	17
Active UC	42	19 (45)	75	3	16	0	23
Total UC	75	35 (47)	116	7	27	1	40
Adenomas	39	12 (31)	149	4	6	2	27
Healthy^a^	55	15 (27)	79402^b^	7	5	3	40
All	185	70 (38)	19279^c^	20	44	6	115

a Healthy individuals have no pathological findings in any of the intestinal mucosal specimens, the “affected colon” is therefore “normal” in these subjects. ^b,c^ When the paired ileum and sigmoid colonic specimens from two healthy patients with exceptionally high B19V-DNA loads (9 × 10^5^ and 1.3 × 10^4^ copies/10^6^ cells for the respective patients) were excluded from the calculation, the mean B19V DNA loads were

b 108 copies/10^6^ cells and

c 110 copies/10^6^ cells.

Intestinal B19V DNA was detected in 35 of 75 (47%) patients with UC, in 16/33 (48%) with inactive, and in 19/42 (45%) with active UC (Table 1). Among B19V-positive UC patients, 11/16 (69%) with inactive UC and 16/19 (84%) with active UC harbored B19V DNA only in the healthy tissue but not in the inflamed colon segment, whereas 4/16 (25%) patients with inactive UC and 3/19 (16%) patients with active UC had B19V DNA in both inflamed colonic and healthy tissues. In contrast, only one (6%) inactive and no (0%) active UC patients had B19V DNA exclusively in the inflamed colon segment (Table 1).

A lower detection rate of B19V DNA was shown in patients with adenoma, 31% (12/39), and in healthy individuals, 27% (15/55; Table 1). The occurrence of B19V DNA (positive or negative) in the patients of each disease group was compared to those of healthy individuals with Fischer’s exact test, but the differences did not reach statistical significance (value of *p* is 0.13 for the malignancy group, 0.38 for adenomas, 0.09 for active UC, and 0.06 for inactive UC).

The occurrence of B19V DNA in either disease-affected colon or healthy tissues was compared intra-individually with Fischer’s exact test. In patients with inactive or active UC, B19V DNA was more often present in healthy tissues than in the diseased colonic tissues, which was statistically significant (Fischer’s exact test, value of *p* < 0.05; Table 2). However, no intestinal tissue preference of B19V persistence was seen in patients with malignancy, adenomas, or in healthy individuals.

**Table 2:** Distribution of B19V DNA in the disease-affected colonic mucosal specimens and normal surrounding colon or ileum of the same patient.

Patients with	Tissue type	B19V+ patients, no.	B19V- patients, no.	Fischer’s exact test, value of *p*
Malignancy	Normal segment	8	8	0.0538
	Colorectal carcinoma^a^	2	14	
Inactive UC	Normal segment	15	18	**0.0148**
	Inactive colon^b^	5	28	
Active UC	Normal segment	19	23	**0.0001**
	Active colon^c^	3	39	
Adenomas	Normal segment	10	29	0.4009
	Colorectal adenoma	6	33	
Healthy subjects	Normal segment	12	43	0.8121
	Colon	10	45	

a Carcinoma adenoma.

b Chronic colitis without activity in the colonic specimens.

c Chronic colitis with active inflammation in the colonic specimens.Numbers in bold are significant.

### B19V DNA Load, Activity, Genotype, and Serology

**Figure 1:**
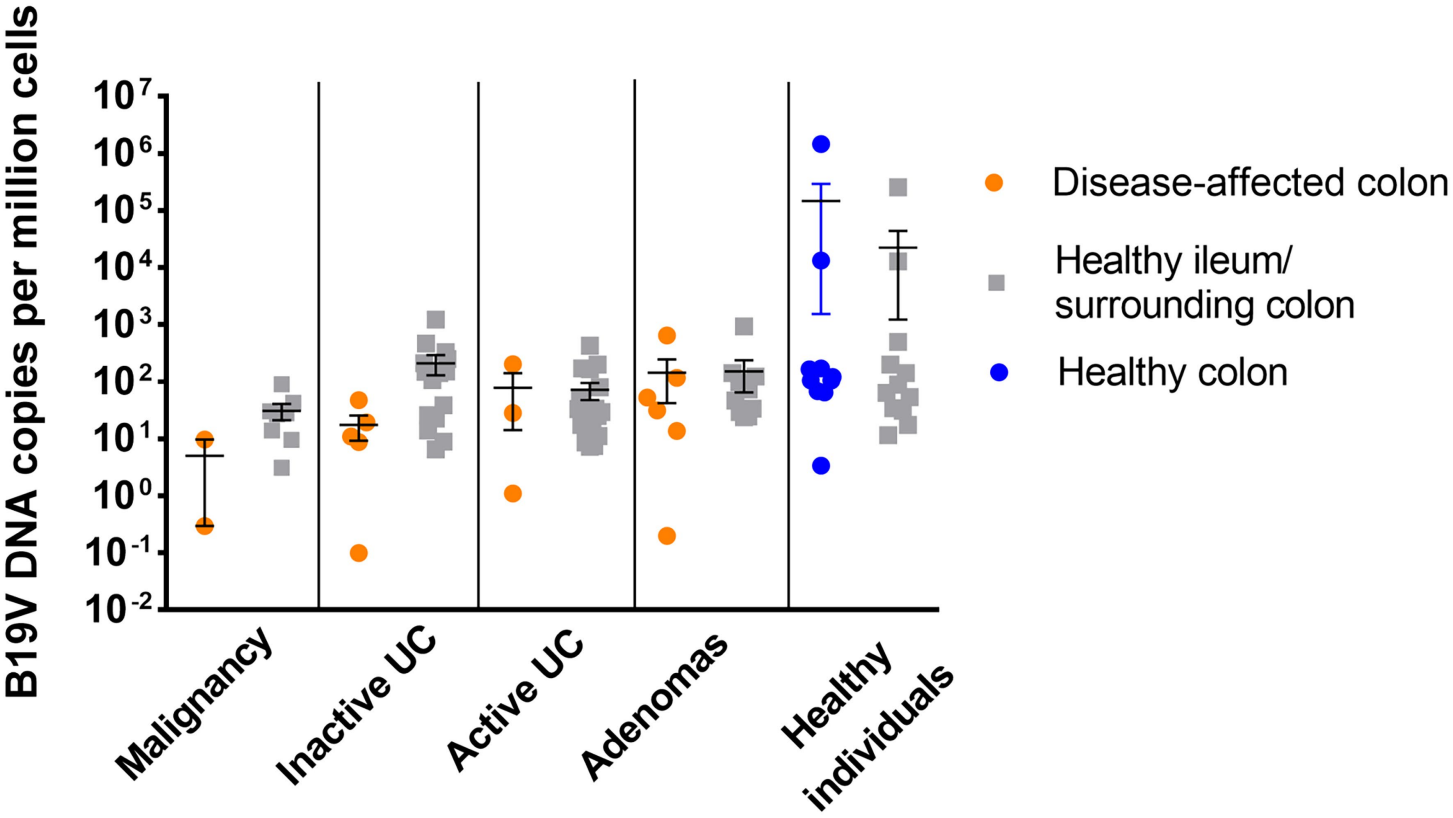
B19V viral load in the disease-affected colonic mucosal specimens (orange) and healthy surrounding colonic specimens or healthy ileum (gray) from patients diagnosed with malignancy, inactive UC, active UC, or adenomas, and in the healthy colonic specimens from healthy individuals (blue). No statistically significant difference in viral load was noted among groups (value of *p* >0.05; one-way ANOVA).

The B19V-DNA loads in intestinal mucosa, overall, were low among all groups (median 4.0 × 10^1^ copies/10^6^ cells; range 0.1 × 10^0^–1.5 × 10^6^ copies/10^6^ cells; Figure 1). Exceptionally high loads of B19V DNA were found in the paired ileum and colon tissues of two healthy individuals, a 72-year-old female and a 78-year-old male, with mean viral loads of 9 × 10^5^ and 1.3 × 10^4^ copies/10^6^ cells for the respective patients. B19V DNA loads were compared among the different disease groups, between the disease-affected and healthy tissues in each group, as well as among disease-affected tissues in the different groups, but no significant differences in viral loads were noted in any comparison (One-way ANOVA and Student’s *t*-test).

B19V activity was determined by measuring virus mRNAs with RT-PCR. The amplification efficiencies of the qPCRs were evaluated with dilution series of the B19V amplicon-containing plasmids, and they ranged from 95 to 105%. Analytical sensitivity of the qPCRs was evaluated with cDNA controls, produced from transfected HEK293 cells; the limit of detection was ≤10 copies/reaction. No B19V mRNA transcripts, either non-spliced *NS1* or spliced *VP*, were detected in the 47 RNAlater stored biopsy samples, including the four high B19V DNA-load samples of the two healthy individuals. Human housekeeping *RPII* mRNAs in the RNA preps were successfully amplified, and the mean quantities were 6.8 × 10^4^ copies/μl (range: 5.8 × 10^3^–1.9 × 10^5^ copies/μl).

Sequence analysis of the qPCR amplicons showed that 57 individuals had B19V genotype 1 and 13 had genotype 2 in the corresponding intestinal mucosa. The two tissue pairs with the highest B19V-DNA loads both harbored genotype 1. All patients harboring B19V genotype 2 were born before 1970 (median age 64.0, range 51.4–86.8), while the patients with genotype 1 varied in age between 20.4 and 82.6, median 50.1.

Of the 13 patients with available serum samples, all had detectable anti-B19V IgG (median OD_450nm_ 3.708), lacked IgM, and had a high ETS ratio (cutoff ≥20), indicating past infection. In line with the serological findings, all these 13 patients had detectable B19V DNA in their intestinal mucosa.

### B19V Persists in Vascular Endothelial Cells and B Lymphocytes

To identify the persistence site of B19V, we applied RISH targeting B19V-*NS1* DNA/mRNA on the FFPE intestinal tissue sections. B19V RISH signals were detected in all biopsy specimens (ileum, as well as ascending, transverse, descending, and sigmoid colon) from the healthy individual who harbored the highest viral loads in this study; 2.6 × 10^5^ and 1.5 × 10^6^ copies/10^6^ cells in the ileum and sigmoid colon, respectively.

**Figure 2:**
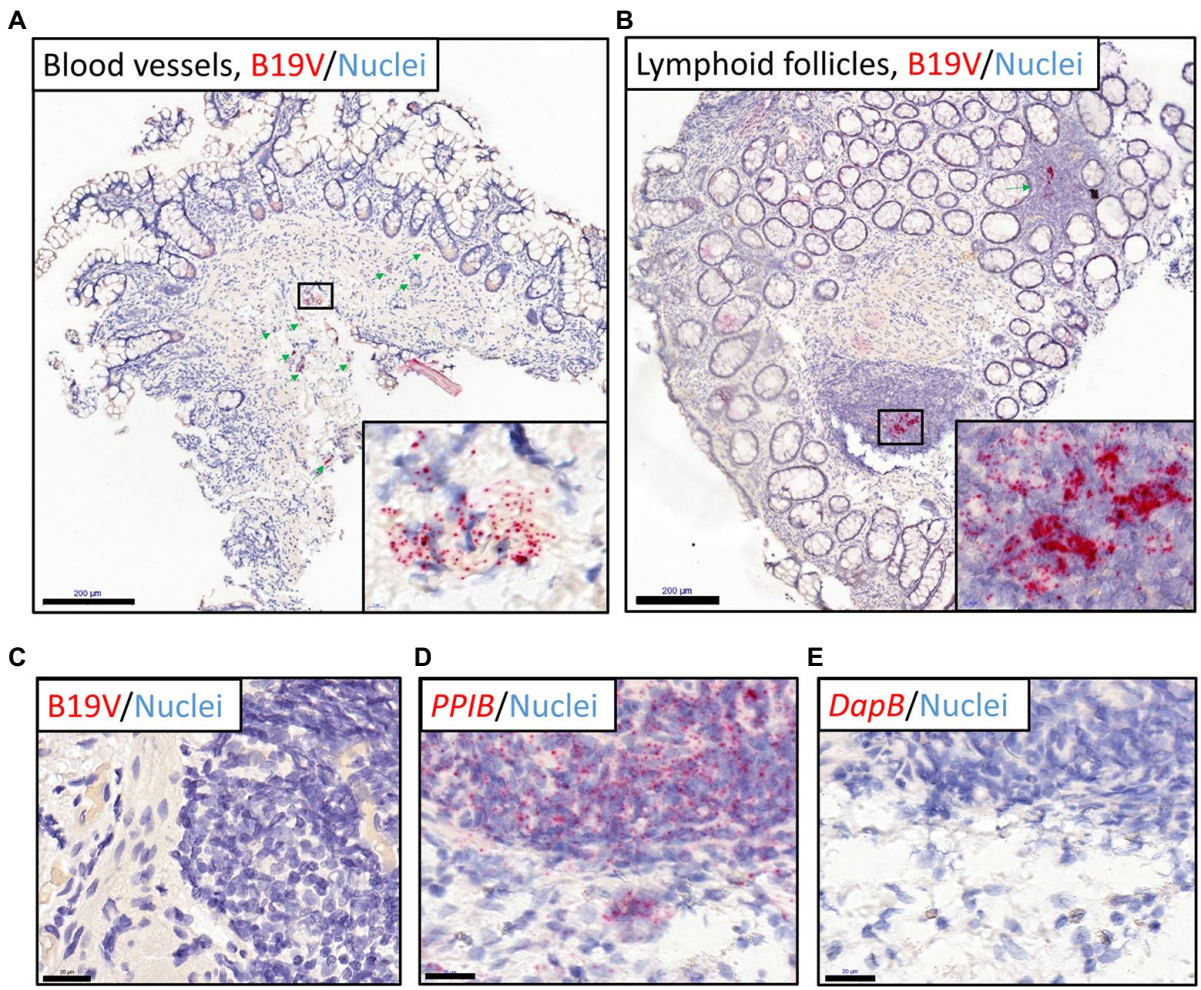
B19V nucleic acid staining of intestinal sections (ileum and colons) from the healthy individual with the highest viral loads by RNAscope ISH (RISH). Red dots represent B19V NS1 or reference gene signals, nuclei are stained blue with hematoxylin. **(A,B)** The high viral load intestinal sections stained with the B19V-NS1 probes showed positive B19V staining in the blood vessels in the healthy ileum **(A)** and lymphoid follicles in the descending colon **(B)**, respectively. Green arrows indicate locations of the dense B19V-signal areas, and higher magnifications of areas with the strongest B19V signals in selected boxes are shown in the bottom-right corners. **(C)** Negative control: the B19V PCR-negative tissue sections showed no B19V staining by RISH. **(D)** Technical positive control: human reference gene *PPIB* RISH showing positive staining of the ascending colon in the above-mentioned individual. **(E)** Technical negative control: probes targeting the bacterial gene *dapB* worked accurately on a consecutive tissue section. Scale bars, 200 μm **(A,B)** and 20 μm **(C-E)**.

Noticeably, in most of these mucosal specimens, strong B19V RISH signals were seen in numerous cells residing mainly in the lymphoid follicles and blood vessels of the intestinal mucosa and submucosa, as verified by an experienced pathologist (Figures 2A,B). More specifically, in the ileum specimen, B19V RISH signals were seen only in blood vessels, as lymphoid follicles were lacking in this tissue section. Unfortunately, FFPE tissue samples were unavailable from the other healthy individual with the second highest B19V DNA load (1.3 × 10^4^ copies/10^6^ cells), while no B19V signals were detected in specimens with lower viral loads (*N* = 5, range 6 × 10^0^–1.2 × 10^3^ copies/10^6^ cells). PCR-negative specimens (*N* = 3) were also RISH negative (Figure 2C). The technical control probes, targeting the human reference gene *PPIB* (red chromogen) and the bacterial *DpIB* gene, worked appropriately (Figures 2D,E).

**Figure 3:**
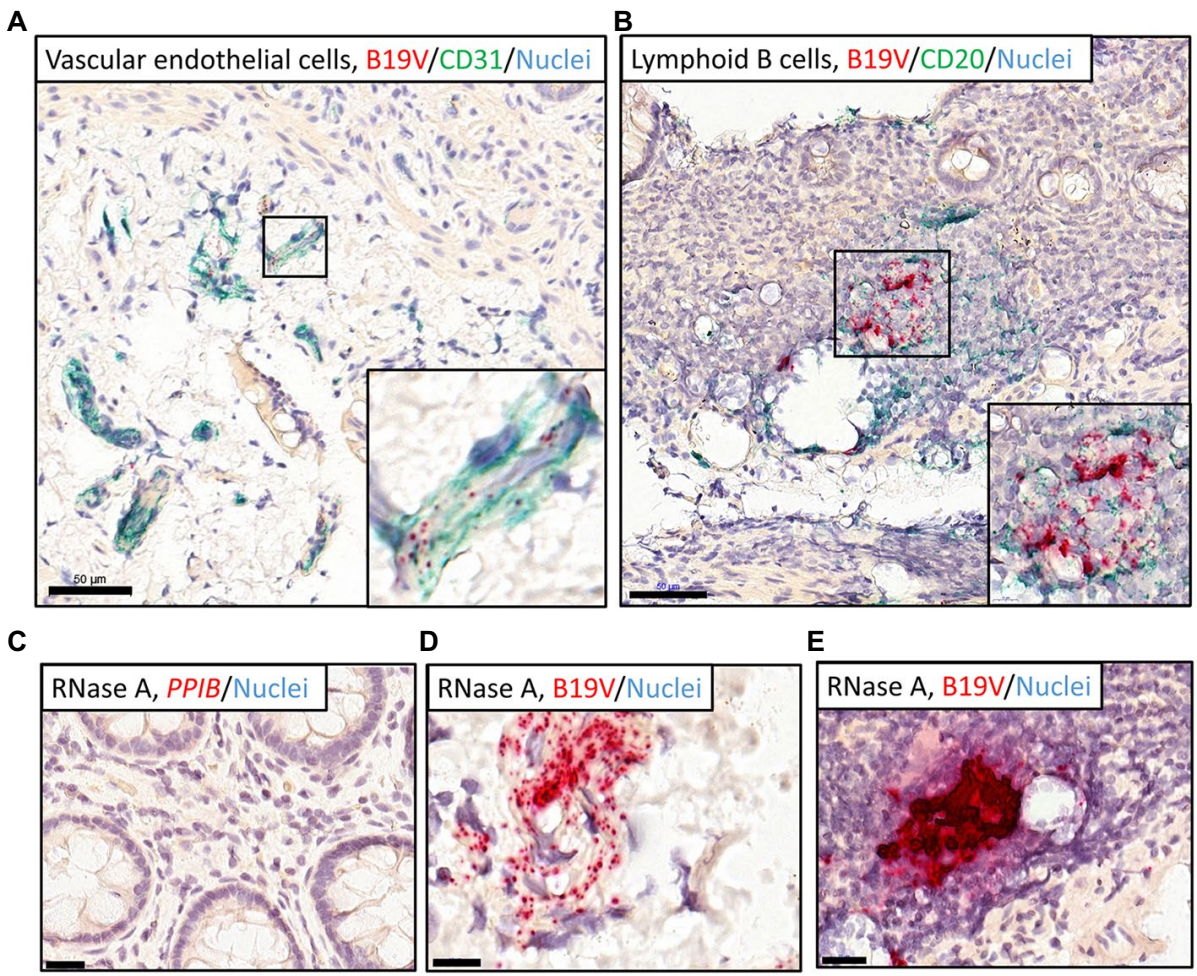
Dual RISH-IHC staining of B19V nucleic acids and cellular markers in the high-load intestinal sections and removal of RNA with RNase A pretreatment. RISH signals appear in red, whereas nuclei are stained blue. **(A,B)** IHC staining of the vascular endothelial cells (CD31; **A**) in the healthy ileum and B lymphocytes (CD20; **B**) in the healthy sigmoid colon following B19V RISH, with cellular markers, stained green. Higher magnifications of the selected areas are shown in the bottom-right corners. **(C)** A RNase A-treated section was stained with the technical positive control probe targeting the human *PPIB* gene. **(D,E)** High-load intestinal sections were pretreated with RNase A before B19V RISH, B19V signals remained in the vascular endothelial cells **(D)** and B lymphocytes **(E)**. Scale bars, 50 μm **(A,B)**, 10 μm **(D)**, and 20 μm **(C,E)**.

Immunostaining for endothelial cell (CD31) and B-cell (CD20) markers by IHC (green chromogen), following B19V RISH (red chromogen), confirmed that the infected cell types were vascular endothelial cells (Figure 3A) and B lymphocytes (Figure 3B).

Cellular RNAs of the tissue sections were eliminated with RNase A treatment, evidenced by no staining by the human reference gene *PPIB* probe (Figure 3C). B19V signals, however, remained visible after RNase A treatment, suggesting the signals were from viral DNA (Figures 3D,E). In B19V antigen IHC, the immunoreactivity could not be interpreted as specific for the B19V qPCR-positive samples, so it was considered negative (not shown).

### Transcriptional Profiling of Intestinal Mucosa With Persistent B19V DNA

**Figure 4:**
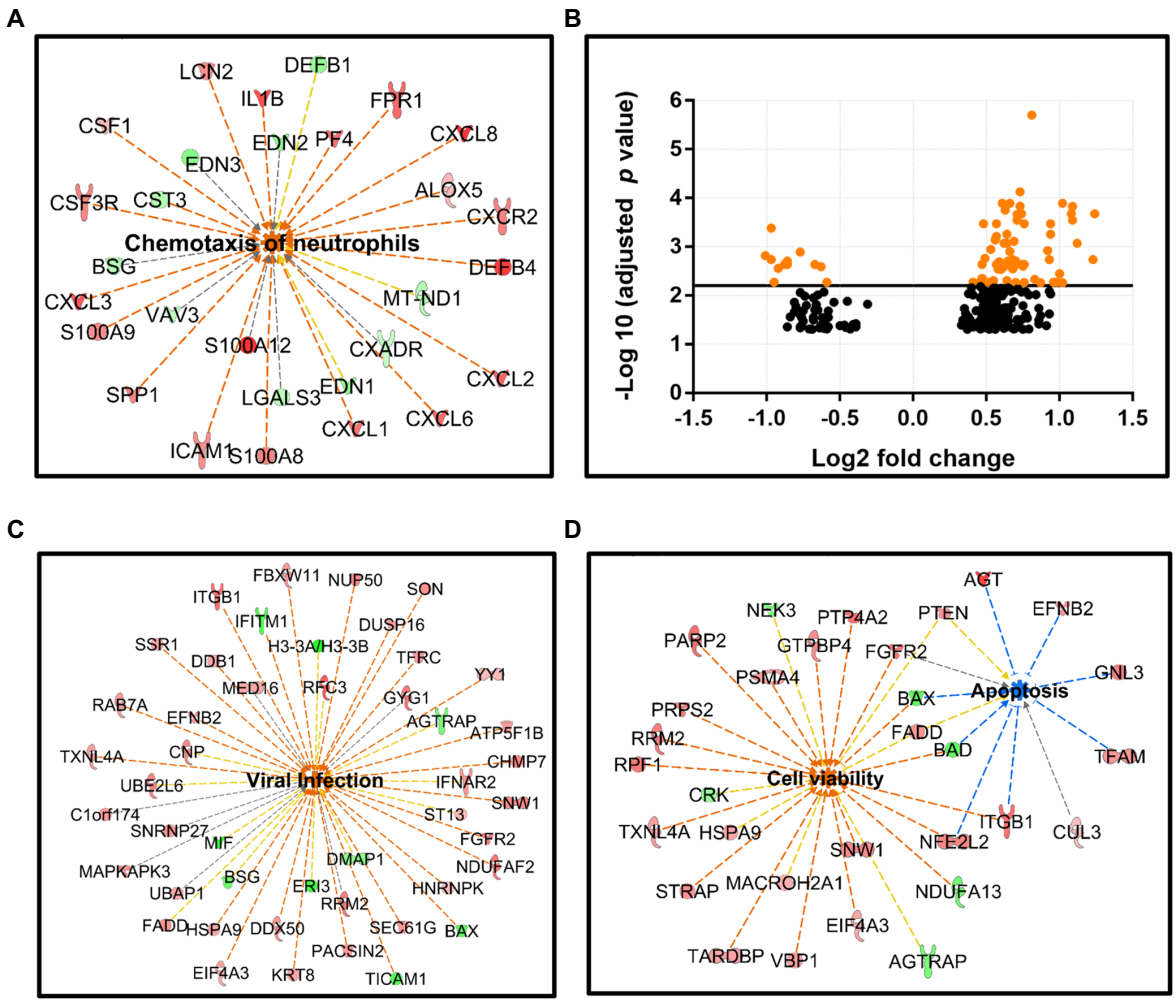
RNA-seq analysis for intestinal mucosal specimens. **(A)** Neutrophil chemotaxis is elevated in the active sigmoid colon tissue compared to healthy sigmoid colon, this pathway involves 29 DEGs: 19 upregulated (red nodes) and 10 downregulated (green nodes), value of *p* 1.6 × 10^−8^, z-score 3.6. **(B)** Scatter plot of the gene expression (272 DEGs) comparing B19V-DNA-positive and -negative ileum, x-axis: Log_2_ fold-change, y-axis: -Log_10_ (adjusted *p* value). DEGs with adjusted *p* values of less than 6.24 × 10^−3^ are shown as orange dots. **(C,D)** Ingenuity pathway on IPA platform (Qiagen) identified different cellular pathways of B19V-altered ileum; 46 DEGs were involved in viral infection **(C)**, and 38 DEGs were involved in cell viability (26 DEGs) or apoptosis (12 DEGs; **D**). DEGs were identified by the Wald test for DEseq2, with Benjamini–Hochberg correction. The *p* values were calculated with Fischer’s exact test (right-tailed), indicating the likelihood of association between a gene set with a process. Z-score indicates the directional effect of the DEGs on a process, z-score > 0 indicates activation, z-score < 0 indicates inhibition, and z-score > 2 or < −2 is considered significant for a given process. Orange to red nodes, upregulated DEGs; green nodes, downregulated DEGs; orange dashed line, DEGs leading to activation; blue dashed line, DEGs leading to inhibition; yellow dashed line, findings inconsistent; gray dashed line, effects unknown; orange center, DEGs leading to activation; and blue center, DEGs leading to inhibition.

To investigate the cellular transcriptome changes induced by persistent B19V infection, we used RNA-seq to compare gene expression in infected and uninfected intestinal mucosa. Our preliminary gene expression analysis showed, not surprisingly, noticeable differences between actively inflamed (*N* = 8) and healthy sigmoid colon tissues (*N* = 8). Compared to the healthy sigmoid colon, inflamed sigmoid colon showed chemotaxis of neutrophils (value of *p* 1.6 × 10^−8^, z-score 3.6, 29 DEGs), featuring upregulated secretion of CXC chemokines (CXCL1, 2, 3, 6, 8) and chemokine receptor (CXCR; Figure 4A), and chemotaxis of leukocytes (value of *p* 5.3 × 10^−8^, z-score 3.4, 50 DEGs, not shown).

The strong immune response in the inflamed sigmoid colon tissue could potentially mask the B19-induced gene expression changes. We therefore selected only healthy ileum mucosa (11 infected and 7 uninfected) from individuals aged 45 to 61 years. Gene expression analysis with DESeq2 package identified 272 DEGs with an adjusted value of *p* < 0.05 (Wald test adjusted by Benjamini–Hochberg), of which 213 genes were upregulated and 59 downregulated (see Supplementary Data Sheet 1). DEGs were visualized with the volcano plot constructed with Log_2_ fold-change (Log_2_ FC) and an adjusted value of *p* (Figure 4B).

To explore the functional networks of the DEGs and biological pathways affected, the DEG datasets with corresponding Log_2_ FC and adjusted value of *p* were uploaded to IPA software (Qiagen) to search for DEG-enriched pathways. As a result, 46 DEGs (9 downregulated and 37 upregulated), generally involved in virus replication, were identified (value of *p* 6.8 × 10^−5^; z-score 2.7; Figure 4C). Noticeably, cell survival was promoted, as shown by the significantly activated cell viability pathway (26 DEGs; 6 downregulated and 20 upregulated, value of *p* 7.3 × 10^−3^; z-score 2.6), and the inhibited apoptosis pathway (12 DEGs; 2 downregulated and 10 upregulated, value of *p* 1.2 × 10^−3^; z-score − 1.8), while the two pathways shared 7 overlapping DEGs, i.e., BAX, BAD, PTEN, FGFR2, NFE2L2, ITGB1, and FADD (Figure 4D). Lastly, 18 DEGs (2 downregulated and 16 upregulated) were enriched in the RNA-splicing network; however, the functional effects of these DEGs were unknown due to their inconsistent contribution to this process (not shown).

### Human Bocaviruses in Intestinal Mucosa

HBoV1, 2, or 3 DNAs were detected by multiplex qPCR in intestinal mucosa of one individual each (1.6%) with ages of 27, 29, and 28 years, respectively, and only in healthy tissues; all exhibited low loads (median, 1.92 × 10^2^ copies/10^6^ cells; range, 2.84 × 10^1^–5.38 × 10^2^ copies/10^6^ cells). No HBoV4 DNA was detected. Specifically, HBoV1 DNA was detected in the healthy ileum of a patient with active UC, whereas HBoV2 DNA was detected in the sigmoid colon, and HBoV3 DNA in both ileum and sigmoid colon, of one healthy individual each. The PCR products were confirmed with Sanger sequencing. No serum samples were available from these individuals. Due to the low viral loads, no HBoV-specific probe signals were observed in RISH.

## Discussion

Parvovirus B19, of the *Erythroparvovirus* genus, causes erythema infectiosum, anemia, arthritis, and hydrops fetalis (Qiu et al., 2017). Associations of B19 with many other diseases have also been studied, including adult myocarditis, rheumatoid arthritis, and other autoimmune diseases; however, the causal role of B19V in these diseases is disputed much due to virus persistence also in healthy individuals (Söderlund-Venermo et al., 2002). B19V DNA remains in several types of tissues of the body for decades after acute infection, which has raised both clinical concerns and interest in the molecular mechanisms and impact of persistence. In this study, we compared the B19V-DNA occurrence in disease-affected and healthy intestinal tissues both within and between individuals and studied the viral persistence sites, host cells, virus activity, and virus-induced transcriptional changes in the tissues. We also elucidated the intestinal persistence of human bocaviruses; HBoV1 being a pathogenic respiratory virus, whereas HBoV2 and HBoV3 are enteric.

The B19V seroprevalence can reach 80% in older adults, whereas the genoprevalence in various tissues have been between 20 and 60% (Söderlund-Venermo et al., 2002; Norja et al., 2006; Adamson-Small et al., 2014; Pyöriä et al., 2017). Our study detected higher B19V DNA occurrence rates in patients with malignancy (50%) or UC (47%) than in healthy individuals (27%), suggesting that B19V may not be a mere bystander. However, the differences in the DNA loads did not reach significance. Both genotypes 1 and 2 were detected, but not genotype 3, which is rare in northern Europe. B19V genotype 2 has not circulated in the population since the beginning of the 1970s (Norja et al., 2006), which explains why all our 13 patients harboring genotype 2 DNA were born before 1970 and had low viral loads.

Although B19V DNA has been detected in many types of tissue, the persistence site and cell types accounting for tissue persistence are poorly studied. With state-of-the-art technology, RNAscope ISH combined with IHC, we localized the sites of B19V persistence to lymphoid follicles, and blood vessels, and identified the host cells to be B lymphocytes and vascular endothelial cells, respectively. Our finding of B19V persistence in B lymphocytes is in line with what has been reported in the synovium and tonsillar lymphoid tissues (Takahashi et al., 1998; Pyöriä et al., 2017). This indicates that also intestinal lymphoid follicles can maintain long-term B19V persistence.

In intrauterine fetal death, B19V DNA and protein have been observed both in erythroid precursors and in CD34-positive capillary endothelial cells of the placental villi (Pasquinelli et al., 2009). B19V DNA has been observed, by ISH, also in arterioles and venules of the adult myocardium in B19V-associated myocarditis (Bültmann et al., 2003; Klingel et al., 2004; Bock et al., 2010). The association between B19 and myocarditis or cardiomyopathy is disputed; however, a recent meta-analysis has shown a significant association between B19V and myocarditis but not cardiomyopathy (Khatami et al., 2022). Our results show that this virus resides in vascular endothelial cells of intestinal tissues of also healthy individuals. Many case reports have observed B19V infection with fetal, pediatric, or adult vascular diseases (Qiu et al., 2017); however, case–control studies could not establish a causal link; and thus, the etiological role of B19V infection in vasculitis remains unresolved (Salvarani et al., 2002; Eden et al., 2003).

The tissue-bound B19V DNA has been speculated to originate from circulating B19V during acute or recent infection. B19V enters the blood stream and causes a systemic acute infection. Viral DNA exists in a long-lasting encapsidated form, allowing sufficient time for virus spread and uptake also in non-permissive cells. An unknown VP1u-recognizing molecule is likely to be the primary cellular receptor for B19V, whereas globoside has been shown to be essential at a post-entry step (Bieri and Ros, 2019; Bieri et al., 2021). Globoside is expressed also in B cells and endothelial cells (Cooling et al., 1995; Müthing et al., 1999; von Kietzell et al., 2014; Zhang et al., 2019). A normal receptor-mediated infection of B19V, or HBoV, in such non-permissive cells is, however, very inefficient. Nevertheless, they can, in the presence of virus-specific antibodies, be more effectively internalized by ADE *via* the Fc receptor or the complement system (Munakata et al., 2006; von Kietzell et al., 2014; Pyöriä et al., 2017; Xu et al., 2021). Inversely, virus-harboring vascular endothelial cells and B lymphocytes of GCs could perhaps spread the virus *via* the blood stream or the lymphatic network to other tissues. B19V virions, or only viral DNA, might also be transported between cells *via* extracellular vesicles, as has been shown for adenovirus (Sims et al., 2014; Ipinmoroti and Matthews, 2020). Moreover, low-load DNaemia can be maintained for more than a year (Musiani et al., 1995; Molenaar-de Backer et al., 2016; Reber et al., 2017), as naked DNA is passively released from damaged tissue or during the natural turnover of tissue cells. Reactivation of persistent B19V is not well documented but some studies have observed viremia of B19V genotype 2 (a virus that no longer circulates in the population but persists in tissues) in immunocompromised patients, suggesting that reactivation can occur (Liefeldt et al., 2005; Grabarczyk et al., 2011; Sterpu et al., 2014).

In our intestinal mucosal tissues, the persisting B19V genomes seem to be quite dormant, evidenced by the generally low viral DNA loads and the absence of viral mRNA. Our study found that B19V mRNA is not only lacking in the healthy intestine, but also in the diseased colonic specimens. A majority of earlier reports of mRNA in tissues have been based on non-quantitative nested PCRs of non-spliced mRNA transcripts, which could potentially be amplified from B19V genomic DNA. In a few studies, B19V capsid proteins have been detected mainly by IHC, which can be non-specific, since non-infected negative control tissues are seldom shown. However, more solid evidence of active replication is lacking. evidence of active replication is lacking. Our B19V IHC did not demonstrate specific capsid protein expression, suggesting that there was no viral protein production, however, low-level expression may have been masked by the unspecific signals. B19V is known to productively infect only cells of the erythroid lineage. Therefore, despite the evidence of B19V mRNA in myocardial endothelial cells, the virus would be unlikely to actively replicate. Nonetheless, even a non-replicating virus may do harm by inducing chronic inflammatory responses and disturbances in cell signaling or proliferation. In general, virus susceptibility studies are difficult to perform in human tissues *in vivo*, but NS1 and VP mRNAs have been detected also in *ex-vivo* primary fibroblast and endothelial cell cultures, however, without productive replication (Zakrzewska et al., 2005).

It is not known how B19V DNA is maintained long term in the tissues in the apparent absence of productive virus replication. Possibly, the virus could integrate its genome into the cellular chromosomal DNA, like another parvovirus, the adeno-associated virus (AAV; Cheung et al., 1980; Smith et al., 2008). The RISH method can detect only non-integrated nucleic acids, while chromosomal DNA is not recognized. Therefore, our clear B19V RISH signals (which were not removed by RNase treatment) prove that there is non-integrated genomic B19V DNA in these cells. However, our positive PCR results may still comprise also integrated viral DNA that is not visible by RISH, which could be another reason why the low-load tissues were RISH negative; however, lower sensitivity remains a more plausible explanation. Intestinal GCs contain abundant B-lineage cells that have been activated by antigens; in addition to the short-lived effector plasma cells, there are also long-lived plasma cells in addition to memory cells (Spencer and Sollid, 2016). It has been reported that the maintenance of rotavirus-specific plasma cells in intestine-associated lymphoid tissues lasts for decades (Spencer and Sollid, 2016; Landsverk et al., 2017); thus, B19V could reside in such long-lived plasma or memory B cells to sustain its persistence. Furthermore, our detection of the non-circulating B19V genotype 2 in intestinal tissues also suggests long-term persistence, as has been shown in tonsillar B cells (Pyöriä et al., 2017).

In transfected erythroid lineage cells, B19V NS1 and the small 11-kDa NS proteins induce apoptosis, choreographed by a variety of caspases (Moffatt et al., 1998; Sol et al., 1999; Chen et al., 2010). Similar findings were shown also with acutely infected EPCs, where apoptosis and apoptotic cell-death pathways were significantly induced by B19V infection, as well as DNA damage response/DNA repair and cell-cycle arrest (Zou et al., 2018). However, comprehensive studies on B19V-modulated cellular processes in persistent human tissues are hitherto very scarce (Qiu et al., 2017). Our study in intestinal mucosal tissues showed for the first time that, instead of inducing apoptosis, long-term B19V tissue persistence rather inhibits apoptosis and activates cell viability, thus promoting cell survival and virus maintenance. Epstein-Barr virus has been shown to exploit a similar mechanism during its latent infection, by upregulating the BHRF1 and BALF1genes, presumably contributing to host-cell survival in order to ensure sufficient virus replication (Fitzsimmons and Kelly, 2017). During acute infection of semi-permissive UT7/Epo-S1 cells (megakaryoblastoid cell line), B19V can induce cellular autophagy to facilitate cell survival (Nakashima et al., 2006), as opposed to apoptosis in EPCs.

For host gene expression studies, we initially performed RNA-seq with the inflamed sigmoid colon tissues, from which we observed strong inflammation responses: upregulation of several chemokines, chemokine receptor CXCR 2, and recruitment of neutrophils and immune cells, which are consistent with findings by others (Danese and Gasbarrini, 2005; Wang et al., 2009). We could unfortunately not use single-cell analysis of our frozen solid tissues. Instead, we used bulk RNA-seq, which measures the average expression of genes across all cell types in the tissue. Hence, any transcriptome changes in a minority of host cells would be diluted by uninfected cells. However, in the healthy ileum tissues, we were still able to observe some biologically relevant pathways to be disturbed by lifelong B19V persistence, such as apoptosis and cell viability. We did not observe, in persistently infected tissues, the same disturbing pathways that are significantly regulated in acutely infected cell cultures, such as cell-cycle arrest or cellular DNA damage and repair, as described (Zou et al., 2018). Possibly due to the dilution effect, B19V-affected genes in our study exhibited small-fold changes, but it could also be the virus’ strategy for reaching equilibrium with the host cell while maintaining its persistent infection. How B19V can modify non-permissive intestinal cells without visible mRNA expression is a very interesting area of research. There could still be some low-level or intermittent viral activity below the detection level, or the cell could react to the unusual foreign DNA, as the linear B19V genome with terminal hairpin structures constitutes.

HBoV2-3 are considered enteric owing to the high DNA prevalence in stool samples; nevertheless, their DNA presence was infrequent in the gut mucosal tissues. The respiratory HBoV1 has been shown in B cells of tonsillar GCs (Xu et al., 2021), which resembles the localization of B19V in intestinal GC B cells; thus, it is easy to speculate that GCs may be the common reservoirs of both B19V and HBoV. The negative RISH results of HBoV in this study may be due to the low viral loads in the tissues, which is in line with our earlier tonsil study that showed a detection limit of approximately10^3^ viral DNA copies per million cells.

In summary, our study provided the first evidence of the tissue sites and cell types accounting for the long-term B19V persistence in intestinal tissues, i.e., B cells of lymphoid follicles and endothelial cells of blood vessels. The long-term B19V persistence in the tissues modulated the cell viability and apoptosis pathways of the host cells. This may have clinical effects in some pre-disposed individuals.

## Data Availability Statement

The datasets presented in this article are not readily available because the raw datasets of RNA-seq contain identifiable human data; thus, we cannot deposit them in a repository for privacy and legal reasons. The B19V-affected DEGs and the clean gene read counts of RNA-seq data of this study can be found in the Supplemental Data Sheet 1 and 2, respectively. Requests to access raw datasets should be directed to maria.soderlund-venermo@helsinki.fi.

## Ethics Statement

The studies involving human participants were reviewed and approved by Ethics Committee of the HUS Helsinki University Hospital (Decision number 553/E6/01). The patients/participants provided their written informed consent to participate in this study.

## Author Contributions

MX, KL, VG, PS, and MS-V conceived and designed the experiments. MX, KL, and VG performed the experiments. MX, KL, TG, VG, JA, PS, and MS-V analyzed the data. KL, VG, AL, TS, PS, and MS-V contributed to the reagents and materials. MX and MS-V: original draft preparation. MX, KL, TG, VG, JA, AL, TS, PS, and MS-V: review and editing. All authors contributed to the article and approved the submitted version.

## Funding

This study was supported by the China Scholarship Council (MX, 201406170014), the Finnish Society for Study of Infectious Diseases (MX), Virustautien Tutkimussäätiö (MX), the Doctoral School in Health Sciences of the University of Helsinki, the Finnish-Norwegian Medical Foundation (MX, 201900123), the Sigrid Jusélius Foundation (MS-V and PS), the Research Funds of the University of Helsinki (MS-V), the Life and Health Medical Support Association (MS-V), and the Swedish Cultural Foundation (MS-V).

## Conflict of Interest

The authors declare that the research was conducted in the absence of any commercial or financial relationships that could be construed as a potential conflict of interest.

## Publisher’s Note

All claims expressed in this article are solely those of the authors and do not necessarily represent those of their affiliated organizations, or those of the publisher, the editors and the reviewers. Any product that may be evaluated in this article, or claim that may be made by its manufacturer, is not guaranteed or endorsed by the publisher.
